# In vitro differentiation of cGMP-grade retinal pigmented epithelium from human embryonic stem cells

**DOI:** 10.1186/s40942-019-0194-7

**Published:** 2019-10-21

**Authors:** Fernando H. Lojudice, Rodrigo A. Brant Fernandes, Francesco Innocenti, Carlos E. Franciozi, Priscila Cristovam, Maurício Maia, Mari C. Sogayar, Rubens Belfort

**Affiliations:** 1 Department of Ophthalmology and Visual Sciences, Federal University of São Paulo, CEP 04021-001 São Paulo, Brazil; 2 Cell and Molecular Therapy Center (NUCEL), Medical School, University of São Paulo, São Paulo, SP 05360-130 Brazil; 3 Department of Biochemistry, Chemistry Institute, University of São Paulo, São Paulo, SP 05508-000 Brazil; 4 Department of Orthopedics and Traumatology, Federal University of São Paulo, São Paulo, 04038-032 Brazil

## Abstract

**Background:**

The World Health Organization (WHO) estimates that the number of individuals who lose their vision due to retinal degeneration is expected to reach 6 million annually in 2020. The retinal degenerative diseases affect the macula, which is responsible for central and detailed vision. Most macular degeneration, i.e., age-related macular degeneration (AMD) develops in the elderly; however, certain hereditary diseases, such as the Stargardt disease, also affect young people. This degeneration begins with loss of retinal pigmented epithelium (RPE) due to formation of drusen (atrophic) or abnormal vessels (exudative). In wet AMD, numerous drugs are available to successful treat the disease; however, no proven therapy currently is available to treat dry AMD or Stargardt. Since its discovery, human embryonic stem cells (hESCs) have been considered a valuable therapeutic tool. Some evidence has shown that transplantation of RPEs differentiated from hESCs cells can result in recovery of both RPE and photoreceptors and prevent visual loss.

**Methods:**

The human embryonic WA-09 stem cell lineage was cultured under current Good Manufacturing Practices (cGMP) conditions using serum-free media and supplements. The colonies were isolated manually and allowed to spontaneously differentiate into RPE cells.

**Results:**

This simple and effective protocol required minimal manipulation and yielded more than 10e8 RPE cells by the end of the differentiation and enrichment processes, with cells exhibiting a cobblestone morphology and displaying cellular markers and a gene expression profile typical of mature RPE cells. Moreover, the differentiated cells displayed phagocytic activity and only a small percentage of the total cells remained positive for the Octamer-binding transcriptions factor 4 (OCT-4) pluripotency cell marker.

**Conclusions:**

These results showed that functional RPE cells can be produced efficiently and suggested the possibility of scaling-up to aim at therapeutic protocols for retinal diseases associated with RPE degeneration.

## Background

Age-related macular degeneration (AMD) is a leading cause of irreversible blindness worldwide [1, 2] that has been estimated to affect more than 8 million people in the United States alone. Despite the introduction of new preventive and treatment therapies, the AMD prevalence should increase by 97% by 2050 [3–5].

Outer retinal degenerative diseases, such as AMD, lead to progressive, irreversible loss of the central visual acuity, with the retinal pigmented epithelium (RPE) the focus of the disease pathophysiology. The role of RPEs is elimination of toxic products resulting from the photoreceptors outer segments metabolism, a function that generally decreases with age.

The advanced forms of AMD are neovascular (wet AMD) and non-neovascular atrophic AMD (dry AMD), both of which are associated with visual acuity loss [5].

A break in Bruch’s membrane may open space for the choroidal vessels to grow into the subretinal space, leading to leakage and subretinal scar formation (wet AMD).

AMD develops when the RPE cannot perform its physiologic role and the metabolic waste from the photoreceptors outer segments begins to accumulate in the subretinal space, under Bruch’s membrane (drusen). This leads to decreased permeability of Bruch’s membrane and reduction of the choroidal vasculature, leading to increased accumulation of waste products and subsequent loss of the underlying RPE, which eventually results in deterioration of the corresponding photoreceptors [6].

Despite the robust development of new therapies and drugs for wet AMD, no proven therapy currently is available to treat dry AMD and outer retinal diseases associated with RPE degeneration.

Some evidence has suggested that transplantation of differentiated RPE cells derived from human embryonic stem cells (hESCs) prevents loss of the photoreceptors and vision in models of rodent macular degeneration [7, 8]. In studies using RCS rats, subretinal transplantation of RPEs derived from hESCs resulted in survival of the photoreceptors near the hESC-RPE implantation site compared with other retinal regions, with vision maintained for longer periods of time compared to controls, and no side effects [8]. This and another safety study [7] have suggested that hESCs may be a potentially safe and inexhaustible source of RPEs to effectively treat various degenerative retinal diseases. Recently, one US group and one South Korean group have reported the safety results and possible biologic activity of RPE cells derived from embryonic cells implanted in patients with dry AMD and Stargardt disease [9–12]. A Japanese group also described for the first time the successful implantation of differentiated RPE cells from the patient’s own reprogrammed induced pluripotent stem (iPS) cells without evidence of side effects [13].

These results, therefore, encouraged other groups to conduct new clinical trials involving RPE cells to treat different types of macular degenerations. However, there is a wide gap between the number of suitable RPE cells obtained from current production systems and the number required for implantation. In addition, commercial and large-scale production of cells for cell therapy protocols requires specific capabilities to develop technologies that will generate safe and effective cell lines [14].

## Methods

### Cell culture

The WA-09 embryonic stem cell lineage [15] was acquired directly from the Wicell Research Institute (WiCell, Madison, WI, USA) and thawed and cultured under current Good Manufacturing Practices (cGMP) conditions at the University of São Paulo (USP) Cell and Molecular Therapy Center (NUCEL) [16]. Pluripotent cell colonies were plated onto hESC-qualified Matrigel (Corning, NY, USA), maintained in mTeSR-1 medium (Stem Cell Technologies, Vancouver BC, Canada) and manually expanded every 4 to 5 days.

### In vitro differentiation and RPE enrichment

Pluripotent WA-09 colonies were plated in six-well plates treated with Matrigel and the medium was renewed daily. To induce differentiation, the mTeSR-1 medium was replaced by serum-free X-VIVO 10 medium (Lonza, Walkersville, MD, USA) supplemented with Normocin (Invitrogen, San Diego, CA, USA), which was changed twice weekly.

For RPE enrichment, the areas with no pigmentation were removed manually from the plates using P200 tips under light phase microscopy. The remaining hESC-RPEs were dissociated with CTS TrypLE Select Enzyme (Invitrogen) for 5 min at 37 °C, passed through a 40-mm nylon cell strainer, counted, and seeded onto Synthemax Surface six-well plates (Corning) at 1.0 × 10e5 viable cells/cm^2^. The hESC-RPEs were maintained in X-VIVO 10 medium and passaged with TrypLE every 30 to 35 days until passage three after enrichment.

### Immunocytochemistry

hESC-RPE monolayers cultured adhered to coverslips were rinsed with Phosphate-buffered saline (DPBS) (Invitrogen) and fixed with 4% paraformaldehyde for 30 min at room temperature, blocked with 5% bovine serum albumin (BSA) and permeabilized with 0.1% Triton X-100 (Sigma-Aldrich, St. Louis, MO) in DPBS for 1 h at room temperature. Primary antibodies, diluted in blocking solution, were incubated with the cells overnight at 4 °C. Coverslips were rinsed three times with DPBS before the secondary antibodies diluted in blocking solution were added and then incubated for 1 h at room temperature. Cells were rinsed three times and 4′,6-diamidino-2-phenylindole (DAPI) (Sigma-Aldrich) was added to the last wash. Slides were mounted with Vecta Shield Antifade Mounting Medium (Vector Laboratories, Burlingame, CA, USA) and images were acquired using the EVOS FL Imaging System (Thermo Fisher Scientific, Waltham, MA, USA). Antibodies: ZO-1 (Thermo Fisher), 1:100 and EZRN-1 (Novus Biologicals), 1:200.

### Quantitative real-time polymerase chain reaction

Quantitative real-time polymerase chain reaction (qRT-PCR) was performed using a ViiA 7 Real-Time PCR System (Thermo Fisher Scientific) with Power SYBR Green (Applied Biosystems, Foster City, CA). Total RNA was isolated using the Illustra RNAspin Mini Kit (GE Healthcare, Little Chalfont, UK), following the manufacturer’s instructions. Oligo-dT primed reverse-transcription was carried out using 2 µg of total RNA in 20 µl of RT reaction with SuperScript III (Invitrogen), followed by qPCR using 3 µl of the fivefold diluted RT reaction in 6 µl of PCR. Transcript levels were normalized to hydroxymethylbilane synthase (HMBS) and expressed as relative abundance using the delta Ct method [17]. Primer pairs (Table 1) were designed to create a 90 to 110 base pair product (Primer Express Software v3.0.1, Thermo Fisher Scientific).

**Table 1:** Sequence of primers used to monitor retinal pigment epithelial differentiation

Primer	Sequence
hBEST1-RT-F	GCCTGCTGAACGAGATGAACA
hBEST1-RT-R	GCTGTACACCGCCACAGTCA
hCRALBP-RT-F	CGAGTGGTCATGCTCTTCAACA
hCRALBP-RT-R	CTGCAAGATCTCATCAAAGGTGA
hEZRIN-RT-F	AATGCCGAAACCAATCAATGT
hEZRIN-RT-R	AGTTGTATTTGGCTGGATTGCA
hHMBS-RT-F	TGGACCTGGTTGTTCACTCCTT
hHMBS-RT-R	CAACAGCATCATGAGGGTTTTC
hPAX6-RT-F	CACCGGTTTCCTCCTTCACA
hPAX6-RT-R	TGGCAGAGCGCTGTAGGTGT
hRPE65-RT-F	CCTCCTGCACAAGTTTGACTTTAA
hRPE65-RT-R	GGAAAGCACAGGTGCCAAATT
hZO1-RT-F	CATCAGATCATTCTGGTCGATCA
hZO1-RT-R	CCGGAGACTGCCATTGCT

### Flow cytometry

Samples of hESC-RPE passage 3 were dissociated with CTS TrypLE Select Enzyme, washed with DPBS and fixed with 4% paraformaldehyde for 30 min at room temperature, blocked with 5% BSA, and permeabilized with 0.1% Triton X-100 in DPBS for 1 h at room temperature. Primary antibodies, diluted in blocking solution, were incubated with the cells for 1 h at room temperature. The cells were rinsed three times, 10 min each with DPBS, then incubated with the secondary antibody diluted in blocking solution for 1 h at room temperature. After incubation period, the cells were washed three times and analyzed in a BD Accuri C6 Flow Cytometer (BD Biosciences, Franklin Lakes, NJ, USA). Antibody: Oct-4 (Cell Signaling), 1:200.

### In vitro phagocytosis assay

hESC-RPE were tested for their phagocytic activity with the pHrodo Red *E. coli* Bioparticles^®^ Conjugate kit (Thermo Scientific), following the manufacturer’s instructions. Briefly, the cells were cultured in 96-wells plates until confluence and then incubated with 100 µl of the above-mentioned particles for 2 h at 37 °C. After the incubation period, the plate was analyzed in a SpectraMax Paradigm Multi-Mode Microplate Reader (Molecular Devices, San Jose, CA, USA) (excitation, 560 nm and emission, 585 nm). Wells with particles but no cells or wells with cells but no particles were used as controls.

## Results

**Fig. 1:**
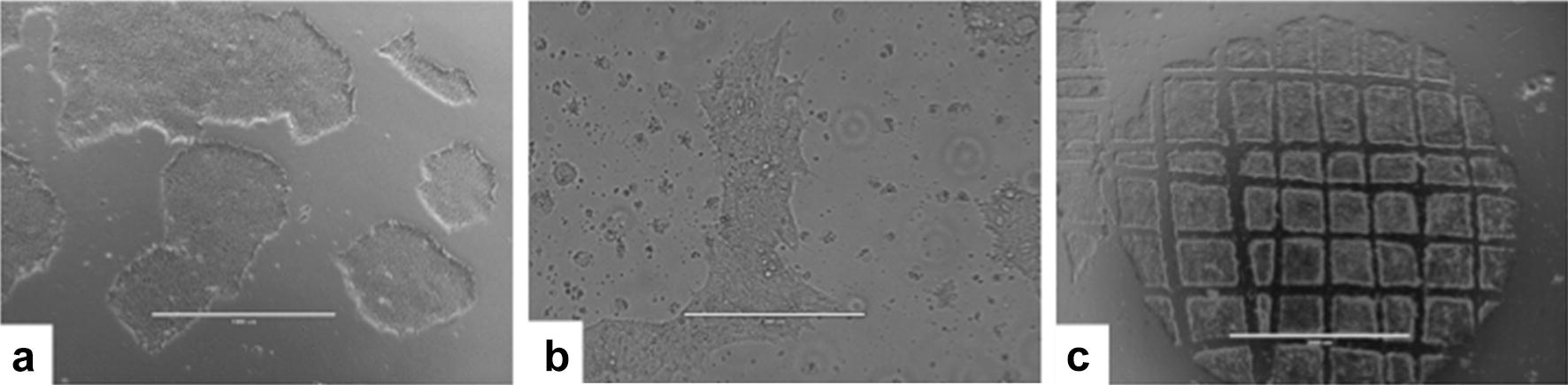
WA09 human embryonic stem cells (hESCs) cultured at the Cell and Molecular Therapy Center. **a** The colonies grown over Matrigel have a homogeneous shape, smooth and regular edges, and no regions with altered color. **b** WA09 hESCs have a high ratio of nucleus to cytoplasm. **c** The colonies are passaged manually by mechanical fragmentation

**Fig. 2:**
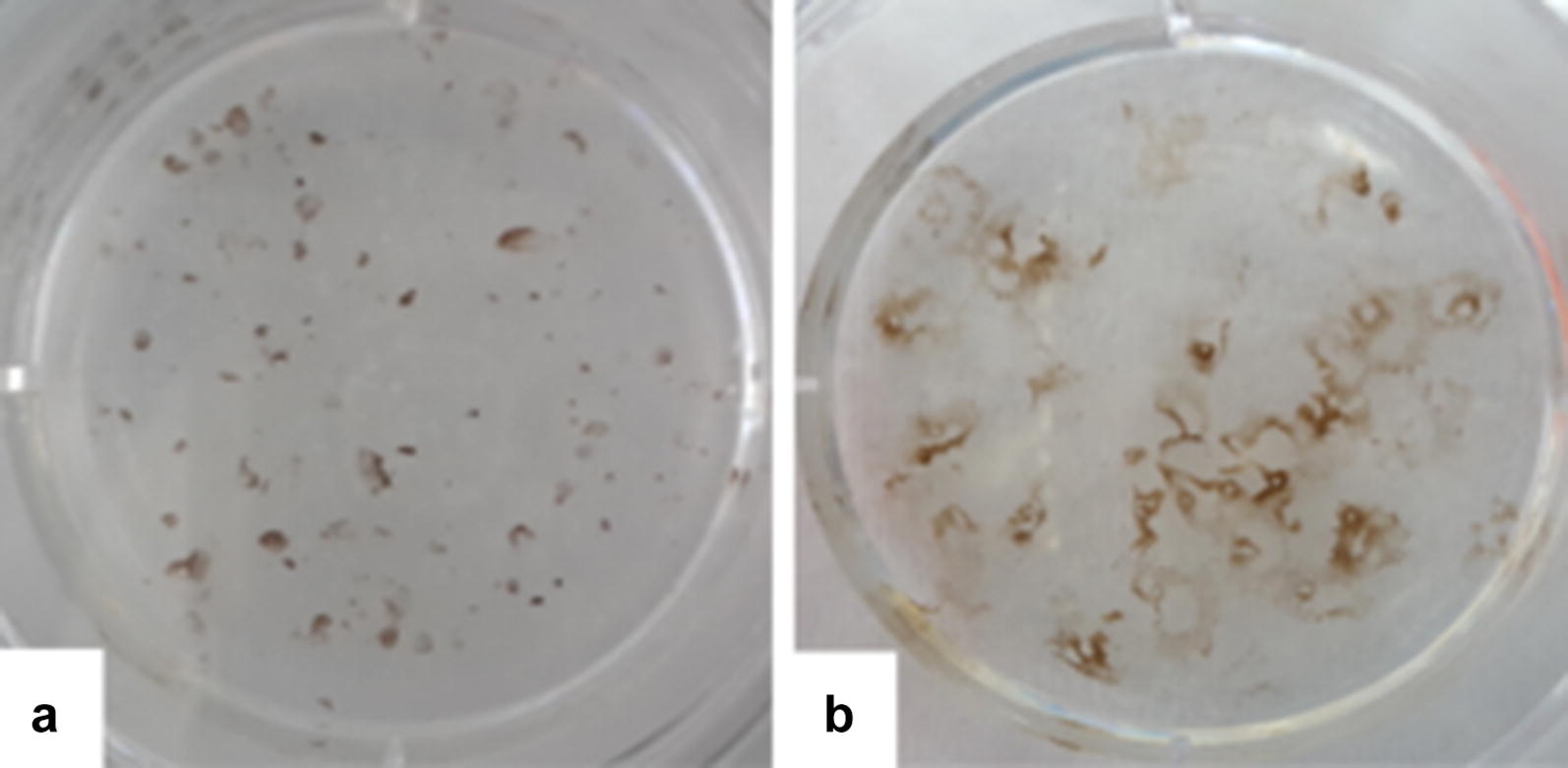
Spontaneous differentiation of WA09. **a** About 30 days after the beginning of the differentiation process, small pigmentated foci are observed in the culture. **b** These pigmented areas expand and darken and, after 120 days of differentiation, the wells are ready for enrichment

The WA09 hESC lineage was thawed and expanded successfully at the NUCEL cGMP facility. These cells do not grow as single cells but, rather, as aggregates in colonies (Fig. 1). These colonies exhibited typical characteristics, i.e., homogeneous shape, smooth and regular edges, absence of regions of altered color, with cells presenting a high ratio of nucleus to cytoplasm (Fig. 2). The cultures were expanded manually by cutting each colony into small fragments, each of which would produce a new colony. The size of each fragment was about 200 × 200 µm (Fig. 1). After fragmentation, the small colonies were harvested gently with a cell scraper. Using these techniques, we maintained our culture growth as pluripotent cells, free from areas of undesired differentiation.

When the cultures reached 70% to 90% confluence, the cells were allowed to spontaneously differentiate into RPE by replacing the mTeSR-1 medium with serum-free X-VIVO 10 medium. Initially, a number of cells detached and died, but the remaining cells resumed growth, with recomposition of the cell monolayer.

At about 30 days after the start of differentiation, small pigmentation foci were observed (Fig. 2). These dark areas expanded and darkened for up 120 days, when the wells were ready for enrichment (Fig. 2).

**Fig. 3:**
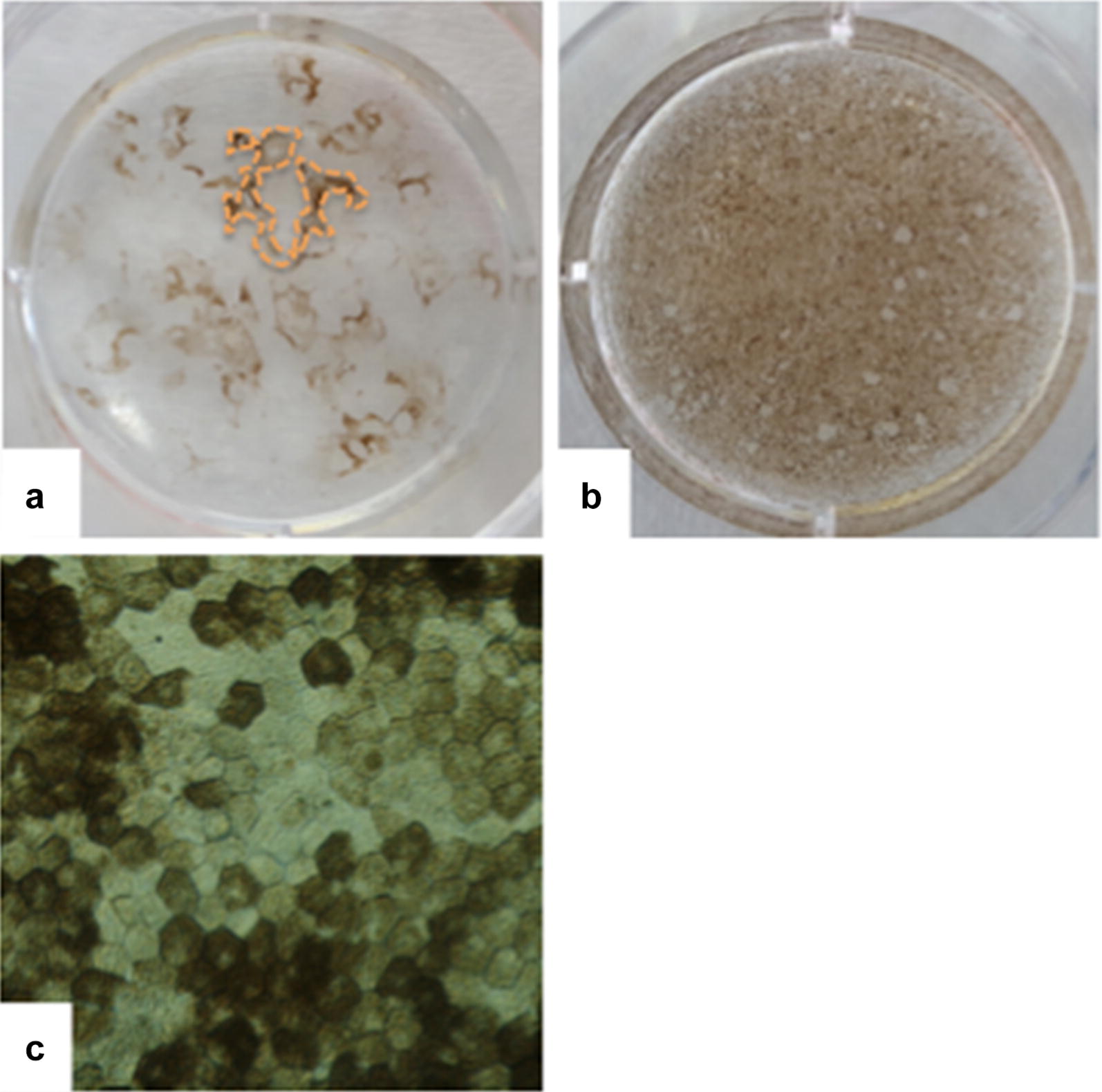
Enrichment for human embryonic stem cell-retinal pigment epithelium (hESC-RPE) cells. When the pigmented regions stopped expanding, the cultures were enriched. **a** The unpigmented areas are scraped off the plate and the remaining cells are passaged enzymatically to a Synthemax-treated well. **b** After up to three rounds of enrichment, the hESC-RPE cultures are homogenous, with purity exceeding 99%. **c** The typical high degree of pigmentation and cobblestone morphology of a hESC-RPE homogenous culture

For enrichment, differentiated pigmented regions were isolated and the areas with no pigmentation were removed manually from the plates using a P200 tip (Fig. 3). The remaining hESC-RPEs were dissociated enzymatically, seeded onto the Synthemax Surface six-well plates and cultured for from 30 to 45 days, when the cells reacquired the epithelial shape and pigmentation and could be subjected to another round of enrichment. Usually, after the first round of enrichment, each well of the hESC-RPE cells yielded 3.10e6 cells, which could then be expanded to three new wells. This procedure was repeated up to three times until a homogenous cultures of hESC-RPE cells were achieved (Fig. 3). After 120 days of differentiation followed by 90 days of enrichment, our process resulted in a very pure population of hESC-RPE cells exhibiting the characteristic cobblestone shape and a high degree of pigmentation (Fig. 3).

**Fig. 4:**
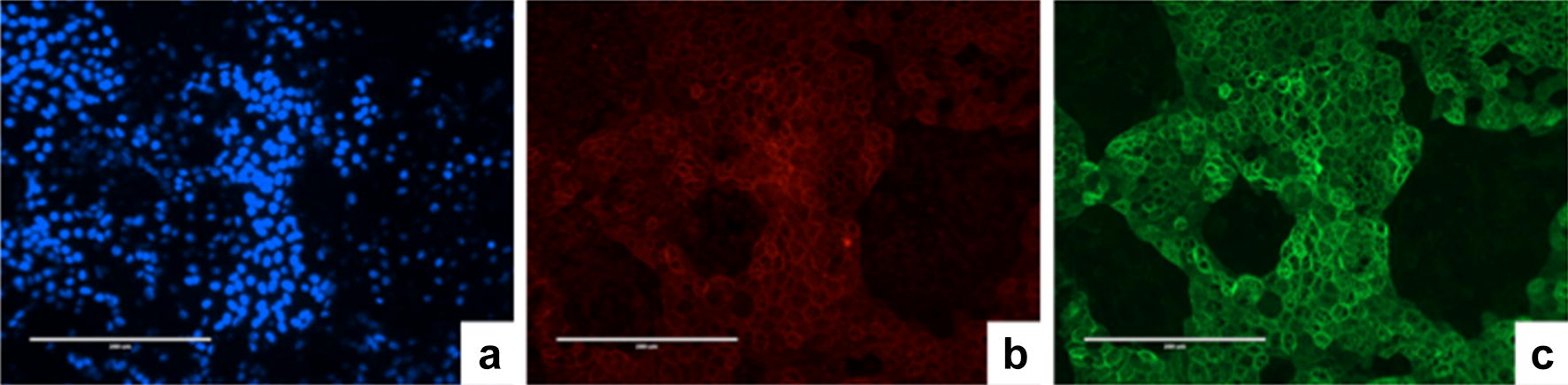
Characterization of human embryonic stem cell-retinal pigment epithelium (hESC-RPE) cells by immunofluorescence. **a** DAPI. Fully differentiated hESC-RPE cells stained for **b** ZO-1 and **c** Ezrin-1. Bar: 200 microns

To characterize the differentiated epithelium, samples were grown onto coverslips treated with Synthemax and then subjected to immunofluorescence labeling against typical mature RPE proteins, i.e., ZO-1 and Ezrin-1 (Fig. 4). Virtually all cells were positive for these RPE markers after differentiation and enrichment.

**Fig. 5:**
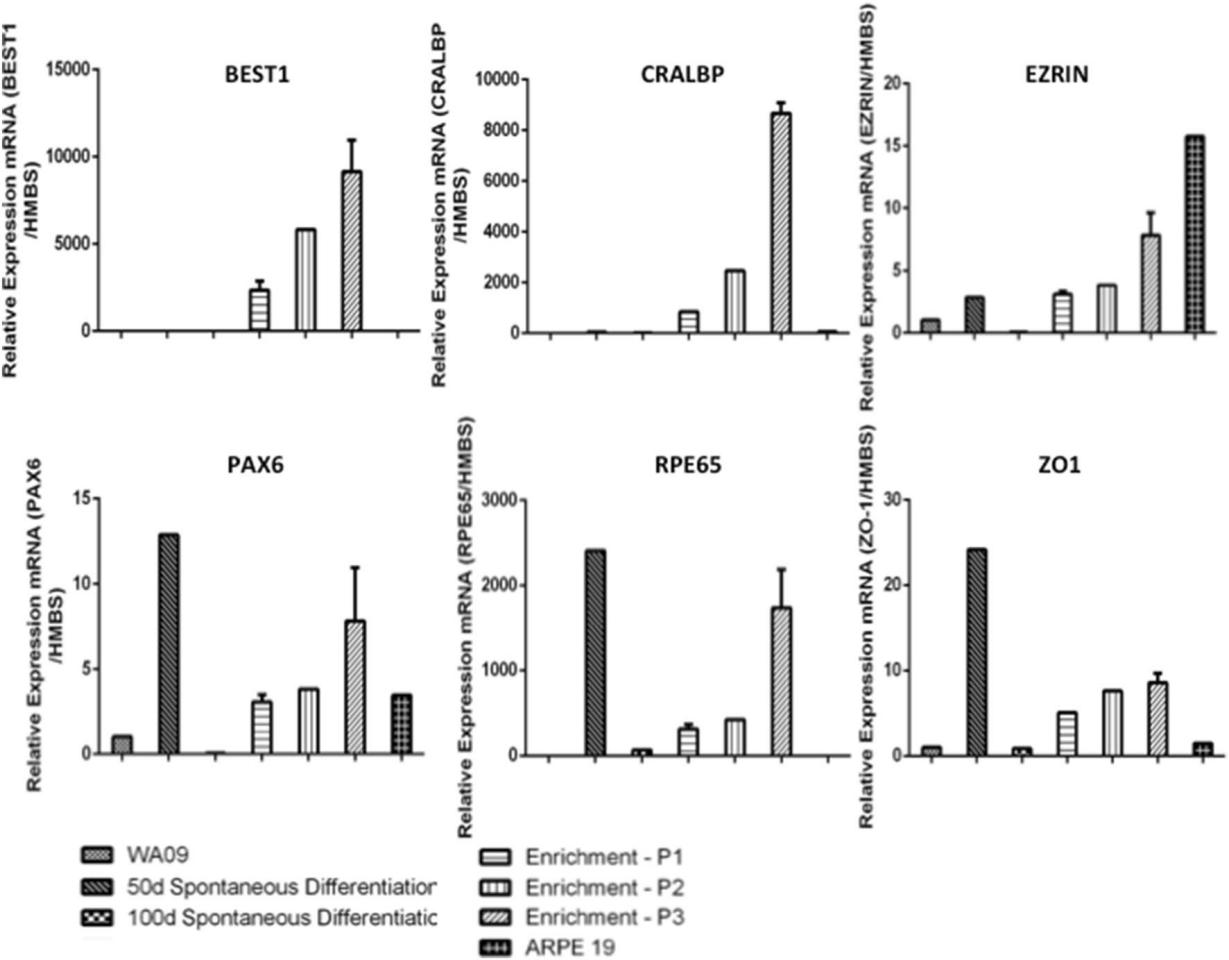
Retinal pigment epithelium (RPE)-related gene expression during human embryonic stem cell differentiation and RPE maturation. Gene expression levels in samples collected at different time points during the differentiation protocol, generated by referencing each gene to hydroxymethylbilane synthase (HMBS) expression levels as an internal control. Undifferentiated WA09 mRNA was used as the comparative sample. The data are expressed as the mean of 2^−DDCT^ ± standard deviation generated by referencing each gene to HMBS. *P* passage

We also observed a progressive increase in the expression of the RNA levels of genes related to development of neuroectoderm and functionally mature RPE cells (Fig. 5), when the enriched cultures were compared to both undifferentiated WA09 cells and even to cultures in different stages of the differentiation process.

**Fig. 6:**
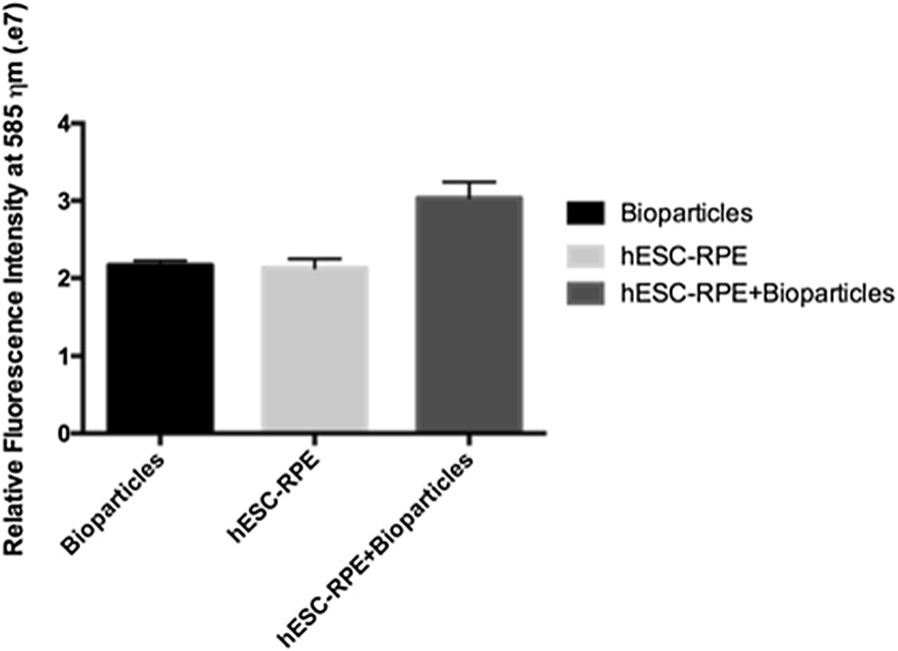
Phagocytic activity of human embryonic stem cell-retinal pigment epithelium (hESC-RPE) cells. The hESC-RPE are incubated in the presence of *E. coli* Bioparticles conjugate, incubated for 2 h, and their internalization capacity analyzed. The fluorescence intensity of both bioparticles and the hESC-RPE cells alone serve as comparative controls

We then tested the functionality of these cells by measuring their ability to undergo phagocytosis. hESC-RPE cell monolayers were incubated in the presence of pHrodo *E. coli* Bioparticles^®^ Conjugate for 2 h at 37 °C. After this incubation period, the samples were analyzed in a microplate reader. Even after a very short period, more particles were internalized and processed by the differentiated cells than by the control samples (Fig. 6).

**Fig. 7:**
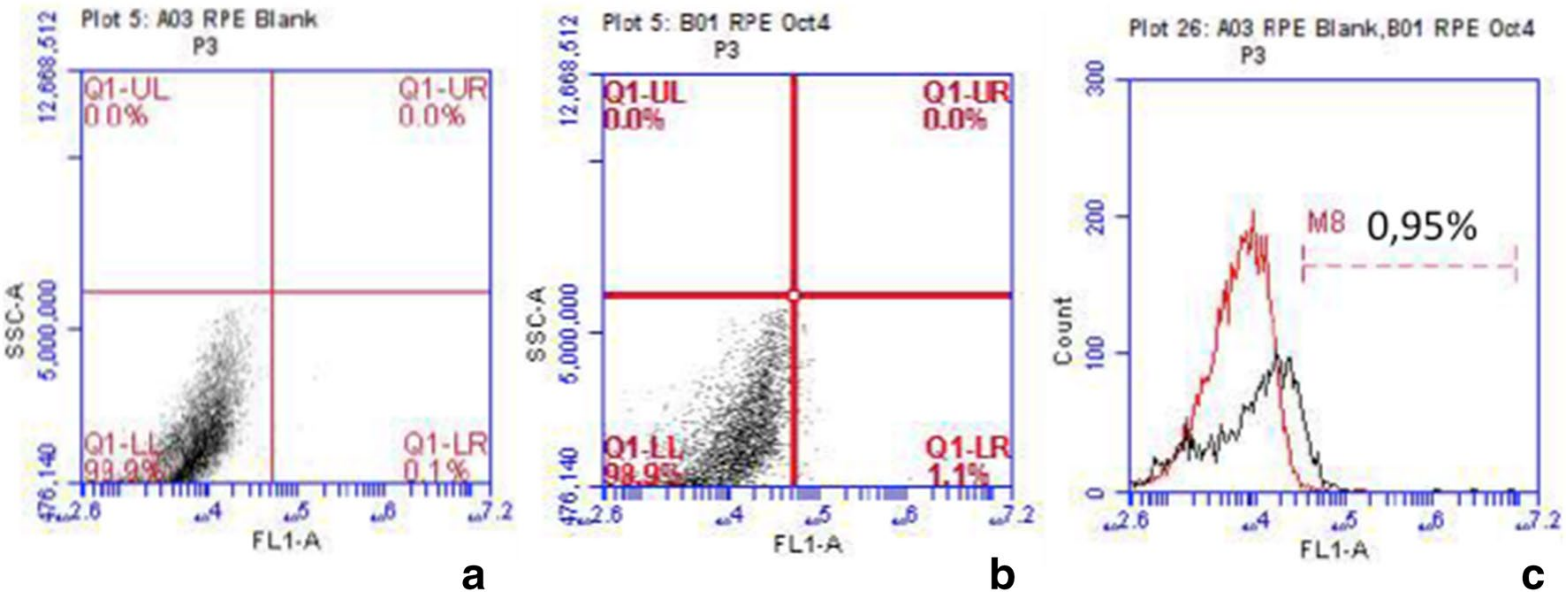
Flow cytometric analysis of human embryonic stem cell-retinal pigment epithelium (hESC-RPE) cells. Samples of differentiated hESC-RPE cells at passage 3 are collected, fixed, incubated in the presence of anti-Octamer-binding transcriptions factor 4 (OCT4) antibody, and analyzed. After 120 days of differentiation plus 90 days of enrichment, less than 2% of the cells are positive for OCT4. **a** The plot shows hESC-RPE cell unstained dispersion (control), **b** The plot shows hESC-RPE cells and anti-OCT4 dispersion. **c** The histogram shows the overlaying of **a**, **b**

Finally, we investigated the presence of undifferentiated cells in the hESC-RPE cell final population. Fluorescence-activated cell-sorting analysis found that only 1.1% of the cells were positive for the Octamer-binding transcriptions factor 4 (*OCT4*) pluripotency marker (Fig. 7).

## Discussion

Blindness severely impacts the quality of human life and is considered a significant economical and social burden worldwide. In the US, the total economic burden was estimated to be US$38.2 billion per year [18]. An even greater impact is evident in individuals younger than 40 years of age.

Since 1998, when James Thomson successfully cultivated hESCs for the first time these cells have been considered a valuable therapeutic tool. Their capacity for self-renewal and differentiation into other cell types make them perfect for use as an unlimited cell source for cell-based therapies [19]. Their therapeutic use has been proposed for treating several degenerative disorders, such as Parkinson’s disease [20], Alzheimer’s disease [21], muscular dystrophies [22], diabetes [23], and retinal degenerative diseases [24, 25]. In 2006, Takahashi and Yamanaka revolutionized the field with the advent of the induced pluripotent stem cells (iPSC) technology [26]. iPSCs are ideal for cell therapy protocols, since they can be generated specifically from each patient, which eliminates tissue rejection and the need for immunosuppression with its resultant side effects. iPSCs also may correct eventual mutations using genetic editing techniques [27]. The therapeutic use of suitable cells derived from human PSCs still poses an important challenge in cell therapy.

To develop RPE replacement therapy, two key challenges must be addressed, i.e., the availability of healthy RPE cells for transplantation and a method of implantation. Several groups have reported that a synthetic substrate seeded with a monolayer of RPE cells may even increase graft survival [28–32].

Although pluripotent cell applications to treat retinal degenerative diseases are in high demand [33–35], it is necessary to elaborate and improve the differentiation protocols (Additional file 1) of these cells into RPE cells under cGMP conditions, using xeno-free or even animal-free compounds [28, 36].

The current study demonstrated that within a few months (4 months maximally), a large number of RPE cells were differentiated from an undifferentiated hESC lineage. This set of techniques favors the development of a cell therapy protocol for AMD that allows generation of a well-defined, well-characterized, readily available structure with no shortage problems. Our results showed that RPE cells differentiated in vitro behave similarly to mature RPE cells, since they express the same proteins displayed by adult RPE cells and also exhibit progressive expression of lineage-specific genes. In addition, using labeled bioparticles, we also showed their phagocytic activity, a characteristic function of these cells. Another important factor to consider is that the number of cells that still maintain pluripotency markers at the end of the protocol is extremely low when compared to similar protocols from another group [37]; this represents great safety in the possible clinical applications of these cells, since the risk of tumorigenesis is strongly related to the number of contaminated pluripotent cells in the culture. In fact, in our pre-clinical experiments, no tumor formation occurred [30].

The clinical translation of RPE cells requires a scalable expansion bioprocess for manufacturing of therapeutically qualified cells. Generally, after the first round of enrichment, each well of hESC-RPE yielded 3.10e6 cells, which can be expanded to three new wells, without diminishing the cellular differentiative status. Therefore, a full six-well plate of hESC-RPE cells at passage 0 can yield up to nine plates at the end of the third round of enrichment or 1.6 × 10e8 cells. According to experimental data from groups already using PSC-derived RPE cells in clinical protocols, this amount of cells would be sufficient to treat more than 1000 degenerated retinas. Based on the growing number of patients diagnosed annually, other methodologies are being proposed to even increase cell production at both lower cost and risk of contamination compared to the conventional monolayer.

In conclusion, our results demonstrated the establishment of a serum-free, feasible, and secure protocol to derive functional RPE cells from human PSCs, with minimal manipulation. Although the costs of production remain high and only a few centers have the technical capacity and trained personnel needed to derive and expand these cells, the RPE implant derived from PSCs offers the greatest promise for treating a disease that still lacks an effective treatment.

## Supplementary information

**Additional file 1: Table S1.** RPE differentiation methods.

## Data Availability

The authors declare that all data supporting the findings of this study are available within the article.
