# Supplementary material for: In vitro differentiation of cGMP-grade retinal pigmented epithelium from human embryonic stem cells
Source: Int J Retina Vitreous. 2019 Oct 21;5:45. doi: 10.1186/s40942-019-0194-7 (PMC6802162; doi:10.1186/s40942-019-0194-7)
Supplement: Supplementary file 1 — Additional file 1: Table S1. RPE differentiation methods. [file 40942_2019_194_MOESM1_ESM.docx]

| ***Reference*** | ***Cell Source*** | ***Differentiation*** | ***Purification/Enrichment*** |
| --- | --- | --- | --- |
| **Lu et al (2009)**  **Schwartz et al (2012)**  **Song et al (2015)** | hESC | - EB - MEM / B27 supplement | - Collagenase type IV + manual isolation - EGM-2 medium - MDBK-MM medium |
| **Buchholz et al (2009)**  **Hu et al (2010)**  **Rowland et al (2012)** | hESC, iPS | - DMEM/F12 + Knckout Serum Replacement | - Mechanically dissected + Trypsin dissociation - DMEM + KSR + FBS + bFGF |
| **Buchholz et al (2013)**  **Leach et al (2015)**  **Foltz et al (2017)** | hESC, iPS | - DMEM/F12 + B27 + N2 + Noggin + Dkk1 + IGF1 + Nicotinamide/3-aminobenzamide + bFGF + Activn A + SU5402 + VIP | - Mechanically dissected + TrypLE Express dissociation - DMEM + FBS |
| **Pennington et al (2015)**  **Thomas et al (2016)**  **Croze et al (2016)** | hESC | - XVIVO10 medium | - Mechanically dissected + TrypLE Express dissociation - XVIVO10 medium |
| **Idelson et al (2009)**  **McGill et al (2017)** | hESC | - EB + 2D - DMEM + KSR + Nicotinamide + Activin A | - Mechanically dissected - DMEM + Nicotinamide |

Table 1. RPE differentiation methods

Lu B, et al. Long-Term Safety and Function of RPE from Human Embryonic Stem Cells in Preclinical Models of Macular Degeneration. Stem Cells. 2009;27:2126–2135

Schwartz SD, et al. Embryonic stem cell trials for macular degeneration: a preliminary report. The Lancet. 2012;379:713–720

Song WK, et al. Treatment of macular degeneration using embryonic stem cell-derived retinal pigment epithelium: preliminary results in Asian patients. Korea trials. Stem Cell Rep. 2015;4:860–872

Buchholz DE, et al. Derivation of functional retinal pigmented epithelium from induced pluripotent stem cells. Stem Cells. 2009;27:2427–2434

Hu Q, Friedrich AM, Johnson LV, Clegg DO. Memory in induced pluripotent stem cells: reprogrammed human retinal-pigmented epithelial cells show tendency for spontaneous redifferentiation. Stem Cells. 2010;28:1981–1991

Rowland TJ, Blaschke AJ, Buchholz DE, et al. Differentiation of human pluripotent stem cells to retinal pigmented epithelium in defined conditions using purified extracellular matrix proteins. J Tissue Eng Regen Med. 2013;7:642-53.

Buchholz DE, Pennington BO, Croze RH, Hinman CR, Coffey PJ, Clegg DO. Rapid and efficient directed differentiation of human pluripotent stem cells into retinal pigmented epithelium. Stem Cells Transl Med. 2013;2(5):384–393.

Leach LL, Clegg DO. Concise Review: Making stem cells retinal: methods for deriving retinal pigment epithelium and implications for patients with ocular disease. Stem Cells. 2015;33:2363–73.

Foltz, L. P. & Clegg, D. O. Rapid, Directed Differentiation of Retinal Pigment Epithelial Cells from Human Embryonic or Induced Pluripotent Stem Cells. *J Vis Exp*, 10.3791/56274 (2017)

Pennington B.O., Clegg D.O., Melkoumian Z.K., Hikita S.T. Defined Culture of Human Embryonic Stem Cells and Xeno-Free Derivation of Retinal Pigmented Epithelial Cells on a Novel, Synthetic Substrate. Stem Cells Transl. Med. 2015;4:165–177

Thomas BB, Zhu D, Zhang L et al. Survival and functionality of hESC‐derived retinal pigment epithelium cells cultured as a monolayer on polymer substrates transplanted in RCS rats. Invest Ophthalmol Vis Sci 2016;57:2877–2887

Croze R. H., Thi W. J., Clegg D. O. ROCK inhibition promotes attachment, proliferation, and wound closure in human embryonic stem cell-derived retinal pigmented epithelium. *Translational Vision Science & Technology*. 2016;5(6):1–7

Idelson M,, Alper R,, Obolensky A,, et al. Directed differentiation of human embryonic stem cells into functional retinal pigment epithelium cells. Cell Stem Cell. 2009; 5: 396–408.

McGill TJ, et al. Long-term efficacy of GMP grade xeno-free hESC-derived RPE cells following transplantation. Transl. Vis. Sci. Technol. 2017;6:17
